# Clinical features and molecular genetics associated with brain metastasis in suspected early-stage non-small cell lung cancer

**DOI:** 10.3389/fonc.2023.1148475

**Published:** 2023-04-17

**Authors:** Kangjoon Kim, Jibeom Lee, Jeong-Yun Lee, Seung Hyun Yong, Eun Young Kim, Ji Ye Jung, Young Ae Kang, Moo Suk Park, Young Sam Kim, Chang-Myung Oh, Sang Hoon Lee

**Affiliations:** ^1^ Division of Pulmonary and Critical Care Medicine, Department of Internal Medicine, Severance Hospital, Yonsei University College of Medicine, Seoul, Republic of Korea; ^2^ Department of Biomedical Science and Engineering, Gwangju Institute of Science and Technology, Gwangju, Republic of Korea

**Keywords:** early stage lung cancer, non-small cell lung cancer, brain metastasis, magnetic resonance imaging, UNC79

## Abstract

**Introduction:**

Regarding whether brain magnetic resonance imaging (MRI) should be routine in patients with suspected early-stage lung cancer, guideline recommendations are inconsistent. Therefore, we performed this study to evaluate the incidence of and risk factors for brain metastasis (BM) in patients with suspected early-stage non-small-cell lung cancer (NSCLC).

**Methods:**

A review of the medical charts of consecutive NSCLC patients diagnosed between January 2006 and May 2020 was performed. We identified 1,382 NSCLC patients with clinical staging of T1/2aN0M0 (excluding BM), and investigated the incidence, clinical predictors, and prognosis of BM in the cohort. We also performed RNA-sequencing differential expression analysis using transcriptome of 8 patients, using DESeq2 package (version 1.32.0) with R (version 4.1.0).

**Results:**

Among 1,382 patients, nine hundred forty-nine patients (68.7%) underwent brain MRI during staging, and 34 patients (3.6%) were shown to have BM. Firth’s bias-reduced logistic regression showed that tumor size (OR 1.056; 95% CI 1.009-1.106, p=0.018) was the only predictor of BM, and pathologic type was not a predictor of BM in our cohort (p>0.05). The median overall survival for patients with brain metastasis was 5.5 years, which is better than previously reported in the literature. RNA-sequencing differential expression analysis revealed the top 10 significantly upregulated genes and top 10 significantly downregulated genes. Among the genes involved in BM, Unc-79 homolog, non-selective sodium leak channel (NALCN) channel complex subunit (UNC79) was the most highly expressed gene in the lung adenocarcinoma tissues from the BM group, and an *in vitro* assay using A549 cells revealed that the NALCN inhibitor suppressed lung cancer cell proliferation and migration.

**Conclusions:**

Given the incidence and favorable outcome of BM in patients with suspected early-stage NSCLC, selective screening with brain MRI may be considered, especially in patients with high-risk features.

## Introduction

Internationally, lung cancer continues to be the leading cause of cancer-related death in men and women (1). Recently, emerging evidence has supported the benefits of lung cancer screening in high-risk patients (2, 3). Early lung cancer screening resulted in a significant relative reduction of 20% in lung cancer mortality and a 7% reduction in total mortality (2). With the aging of the population and introduction of screening for high-risk individuals, the incidence of clinical stage I non-small-cell lung cancer (NSCLC) is likely to increase accordingly (4).

An increasing number of patients with early-detected lung cancer can raise clinical questions about what needs to be done to adequately staging these patients. Because unidentified extrathoracic metastasis may result in improper treatment and poor prognosis, detection of distant metastasis before initiation of treatment is crucial. Therefore, all patients with pathologically proven primary lung cancer should undergo a positron emission tomography (PET) scan (5). PET is particularly useful in M staging of NSCLC and can replace the use of bone scintigraphy (6), but it is limited in the assessment of brain metastasis (BM) compared with magnetic resonance imaging (MRI) (7).

Regarding whether brain imaging with MRI should be routine in patients with suspected early-stage lung cancer, guideline recommendations are inconsistent. The National Comprehensive Cancer Network (NCCN) guidelines recommend brain MRI in patients with stage II to IV NSCLC, suggesting that it is optional in patients with stage IB NSCLC (8). The National Institute of Health and Care Excellence (NICE) recommends brain MRI in patients with stage III NSCLC and suggests that brain imaging tests should not be performed in clinical stage I patients (9). On the other hand, the recommendations of the British Thoracic Society are that all patients being considered for surgery with curative intent should receive routine brain imaging, regardless of clinical stage (10).

Milano et al. investigated the incidence of BM in patients with node-negative NSCLC with the Surveillance, Epidemiology, and End Results (SEER) registry data of 49,495 participants with stage T1-3N0 disease (11). In that study, the incidence of BM in patients with T1N0 clinical staging was revealed to be approximately 3%. However, the previous study did not exclude patients with known distant metastasis other than BM, and data regarding the diagnostic method used for staging were not available.

Due to the limited number of high-quality studies on BMs in patients with clinical stage I lung cancer, the level of evidence to support current recommendations is still relatively low, and it is controversial whether brain MRI should be performed routinely in patients with stage I lung cancer.

Given these considerations, we performed the current study to evaluate the actual incidence of BM confirmed by MRI in patients with suspected stage I NSCLC (radiological T1N0 or T2aN0 NSCLC, no distant metastasis) based on CT and PET scan. Furthermore, we also investigated the particular risk factors for BM in that population. Specifically, we aimed to identify clinical parameters predicting BM at first stage of study, and then we perform RNA-sequencing differential expression analysis using transcriptome of subset of patients with BM and matched controls.

## Methods

### Patients

This retrospective study examined patients who were diagnosed with NSCLC in Severance Hospital, a university-affiliated tertiary referral hospital in South Korea, between January 2006 and May 2020.

**Figure 1:**
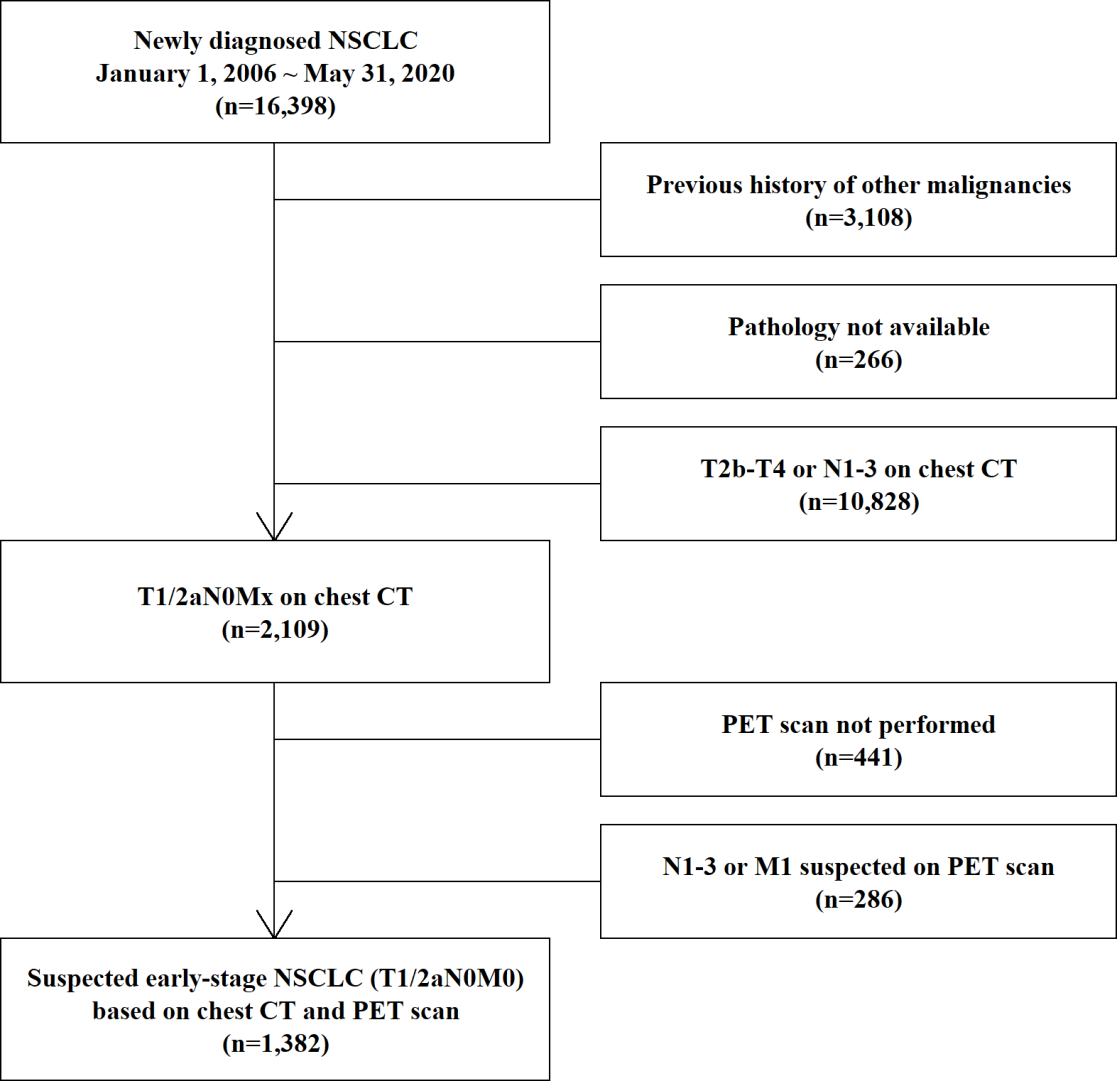
Process of selection of the study population. NSCLC, non-small-cell lung cancer; CT, computed tomography; PET, positron emission tomography; MRI, magnetic resonance imaging.

Among the 16,398 newly diagnosed NSCLC patients, we excluded 3,108 patients with a previous cancer history because asynchronous double cancer could obscure the true origin of the BM. According to the selection process (**Figure 1**), we finally selected 1,382 patients with early-stage lung cancer (T1/2aN0M0) excluding status of BM, based on chest CT, PET scan, and bronchoscopy data. The TNM stage classification was based on the 8th edition of the IASLC staging system (12).

The study was approved by the Ethics Review Committee of Severance Hospital (IRB No. 4-2020-1306), and the need for informed consent was waived by the committee due to the retrospective nature of the study.

### Clinical data collection

We collected the patients’ demographic information, smoking history, radiology and pathology reports, and history of diagnostic evaluation and treatment from their electronic medical records.

All diagnoses of NSCLC were confirmed by a pathology report of the biopsied specimen, and information on the pathologic type was also recorded for analysis. Primary tumor sizes were measured by two experienced radiologists with thoracic specialization. In the case of subsolid lesions, the solid portion size was measured and recorded for analysis, with reference to the 8th edition of the IASLC staging system.

BMs were confirmed retrospectively by screening databases of radiology reports of gadolinium contrast-enhanced MRI of the brain, which were made by neuroradiologists at the time of diagnosis. When the brain lesion was equivocal for BM, multidisciplinary team diagnosis and review of serial brain MRI images confirmed the diagnosis of BM.

### RNA-sequencing procedures

After identification of patients with BM, all available paraffin-embedded lung tumor samples of the patients were retrieved from tissue repository in our institution. The samples were subjected to quality check process during library preparation for RNA sequencing. Among the samples, total 7 samples were passed the quality check and proceeded to RNA sequencing.

Paired controls without BM were selected for molecular analysis, according to five factors: sex, age, tumor size, pathologic type and known genetic mutation. When tissue with identical age or tumor size were not available for the controls, the closest number of age or tumor size was chosen for control selection. Quality check and RNA sequencing procedure were identical to the sample of BM group. All BM samples were matched to corresponding control samples.

Sections (5 µm thick) from paraffin-embedded lung tumor tissues were dewaxed in xylene for 10 min, washed in 100%, 90% and 80% ethanol and rehydrated in distilled water. Unstained sections were analyzed under a microscope to scrape tumor areas with at least 70% tumor cells. DNA and RNA were isolated using the AllPrep DNA/RNA FFPE Kit (Qiagen, Hilden, Germany). Isolation was performed according to the manufacturer’s instructions. Sequencing was performed with an Illumina NextSeq550 (Illumina Inc., CA, USA) after library preparation with Illumina RNA Prep with Enrichment (Illumina Inc., CA, USA) according to the manufacturer’s protocol.

### RNA-sequencing data analysis

RNA-sequencing data were analyzed with differential expression analysis and pathway enrichment analysis in terms of gene ontology (GO) and Kyoto Encyclopedia of Genes and Genomes (KEGG). RNA-sequencing differential expression analysis were performed using DESeq2 package (version 1.32.0) with R (version 4.1.0), and the median-of-ratios method was used to normalize for RNA composition and sequencing depth (13). Detailed descriptions of the analysis protocol are presented in the **Supplementary Materials** (**Supplementary Methods** section).

### *In vitro* assay using A549 cells with NALCN inhibitor

To investigate the role of the Na^+^ leak channel, non-selective (NALCN) in lung cancer proliferation and metastasis, we performed an *in vitro* assay using A549 cells with the NALCN inhibitor, L-703,606 (14). Detailed descriptions of the cell culture and cell proliferation assay methods are presented in the **Supplementary Materials** (**Supplementary Methods** section).

### Statistical analysis

Descriptive variables are summarized with means, medians, standard deviations, interquartile ranges, and proportions and were compared according to the dependent variables of interest using Fisher’s exact test, T test, or Mann–Whitney U test. To determine the risk factors for BM, we used Firth’s bias-reduced logistic regression to reduce the bias caused by rare events. Fisher’s exact test with the minimum p value approach was used to determine the optimal cutoff value in tumor size for predicting BM. Fisher’s exact test was adopted for this procedure due to small sample of events and unbalanced data (15). A *p value <*0.05 was considered significant for all analyses.

Data analysis was performed using SPSS version 26 (IBM Corp. Released 2019. Armonk, NY, USA) and R (version 4.0.3; R Foundation for Statistical Computing, Vienna, Austria).

## Results

### Clinical characteristics of the study population

**Table 1** shows the clinical characteristics of the study population. Six hundred fifty-one of the 1,382 patients (47.1%) were women, 731 (52.9%) were men, and the mean age was 64.3 ± 10.4 years. Six hundred seventy-six patients (48.9%) were ever-smokers. Adenocarcinoma was the most common pathologic type (82.3%), followed by squamous cell carcinoma (16.9%) and large cell carcinoma (0.4%). The mean tumor size was 22.0 ± 8.0 mm. Nine hundred forty-nine patients (68.7%) underwent brain MRI during staging. Patients with advanced age or larger tumor sizes were more prone to undergo brain MRI during the staging workup (p <0.05).

**Table 1:** Clinical characteristics of the study participants.

	Total		Brain MRI				p value
			Performed		Not performed		
Patients, n	1,382		949 (%)		433		
Female (%)	651	(47.1)	450	(47.4)	201	(46.4)	0.771
Age (years)	64.3	± 10.4	64.8	± 10.6	63.2	± 10.1	0.007
Ever-smoker (%)	676	(48.9)	470	(49.5)	206	(47.6)	0.524
Pathologic type							0.119
ADC	1,137	(82.3)	777	(81.9)	360	(83.1)	
SqCC	233	(16.9)	167	(17.6)	66	(15.2)	
LCC	6	(0.4)	3	(0.3)	3	(0.7)	
Others^*^	6	(0.4)	2	(0.2)	4	(0.9)	
Primary tumor size, mm	22.0	± 8.0	23.1	± 7.7	19.8	± 8.1	<0.001

^*^ Includes mucoepidermoid carcinoma (n=2), sarcomatoid carcinoma (n=1), and non-small cell carcinoma not otherwise specified (n=3).

MRI, magnetic resonance imaging; ADC, adenocarcinoma; SqCC, squamous cell carcinoma; LCC, large-cell carcinoma.

### Incidence of and clinical risk factors for BM

Among the 949 patients who underwent contrast-enhanced brain MRI, 34 patients (3.6%) were revealed to have BM and were diagnosed with stage IV NSCLC. Group comparisons of the patients according to the occurrence of BM showed no statistically significant difference between the two groups in sex, age, smoking history, or pathologic type, but the mean tumor size was larger in the metastasis group (p >0.05) (**Table 2**).

**Table 2:** Group comparison of participants who underwent brain MRI according to the presence of isolated brain metastasis at diagnosis.

	Stage IV NSCLC BM-positive		Stage I NSCLC BM-negative		*p value*
Patients, n	34		915		
Sex, female	17	(50.0)	433	(47.3)	0.862
Age, y	62.5	(56-75)	65.0	(58-73)	0.894
Ever-smoker	15	(44.1)	455	(49.7)	0.602
Pathologic type					0.850
ADC	29	(85.3)	748	(81.7)	
SqCC	5	(14.7)	162	(17.7)	
LCC	0	(0.0)	3	(0.3)	
Others^*^	0	(0.0)	2	(0.2)	
Tumor size, mm	25.5	(20-35)	23.0	(18-28)	0.031

Categorical data are presented as numbers (%) and tested with Fisher’s exact test; continuous data are presented as medians (IQRs) and tested with the Mann–Whitney U test.

^*^ Includes mucoepidermoid carcinoma (n=2), sarcomatoid carcinoma (n=1), and non-small cell carcinoma not otherwise specified (n=3).

MRI, magnetic resonance imaging; NSCLC, non-small-cell lung cancer; ADC, adenocarcinoma; SqCC, squamous cell carcinoma; LCC, large-cell carcinoma.

**Figure 2:**
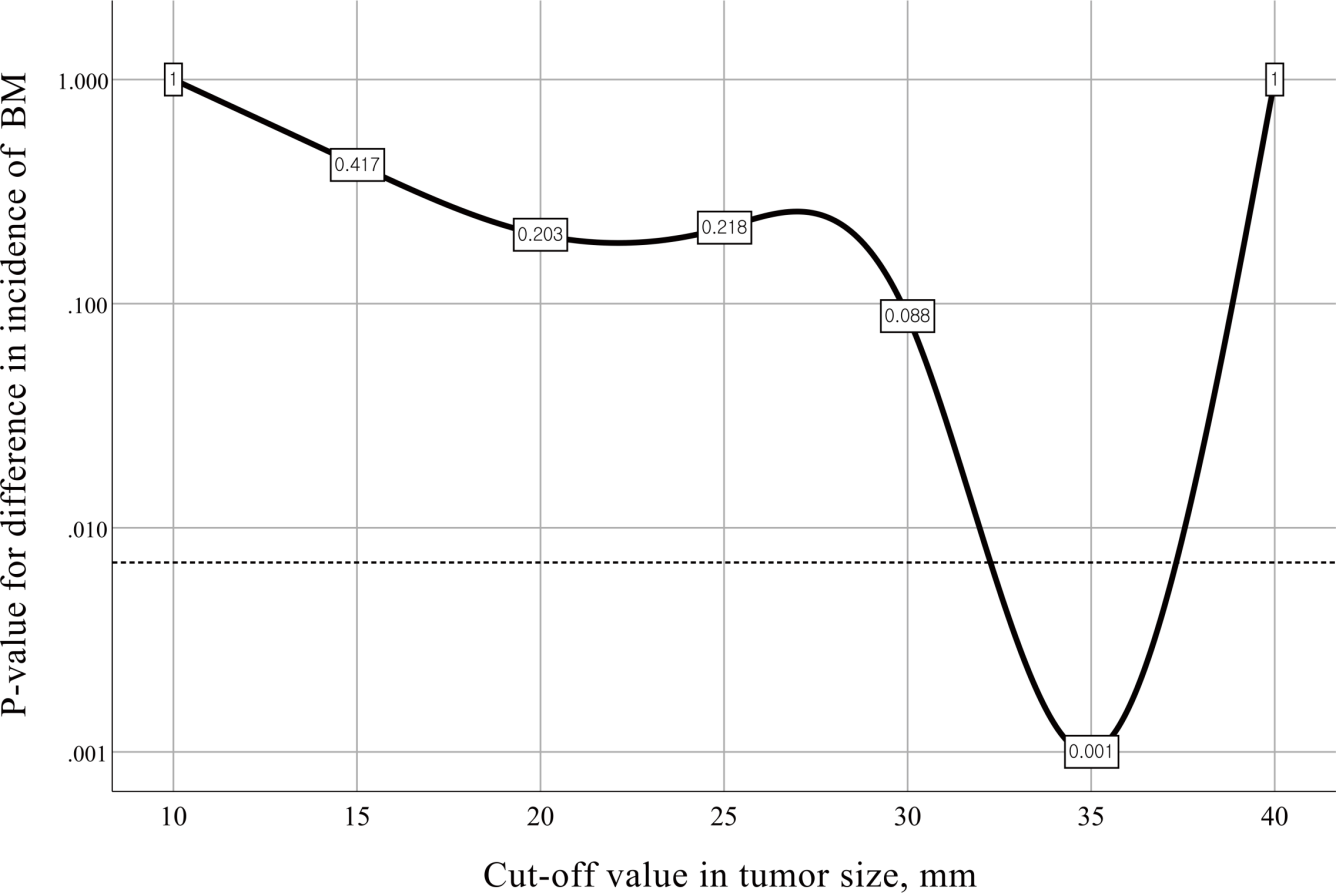
Charts for different cutoff points with corresponding p values for difference in the incidence of brain metastasis. The dotted line represents a Bonferroni-adjusted significance level of 0.007. BM, brain metastasis.

**Figure 3:**
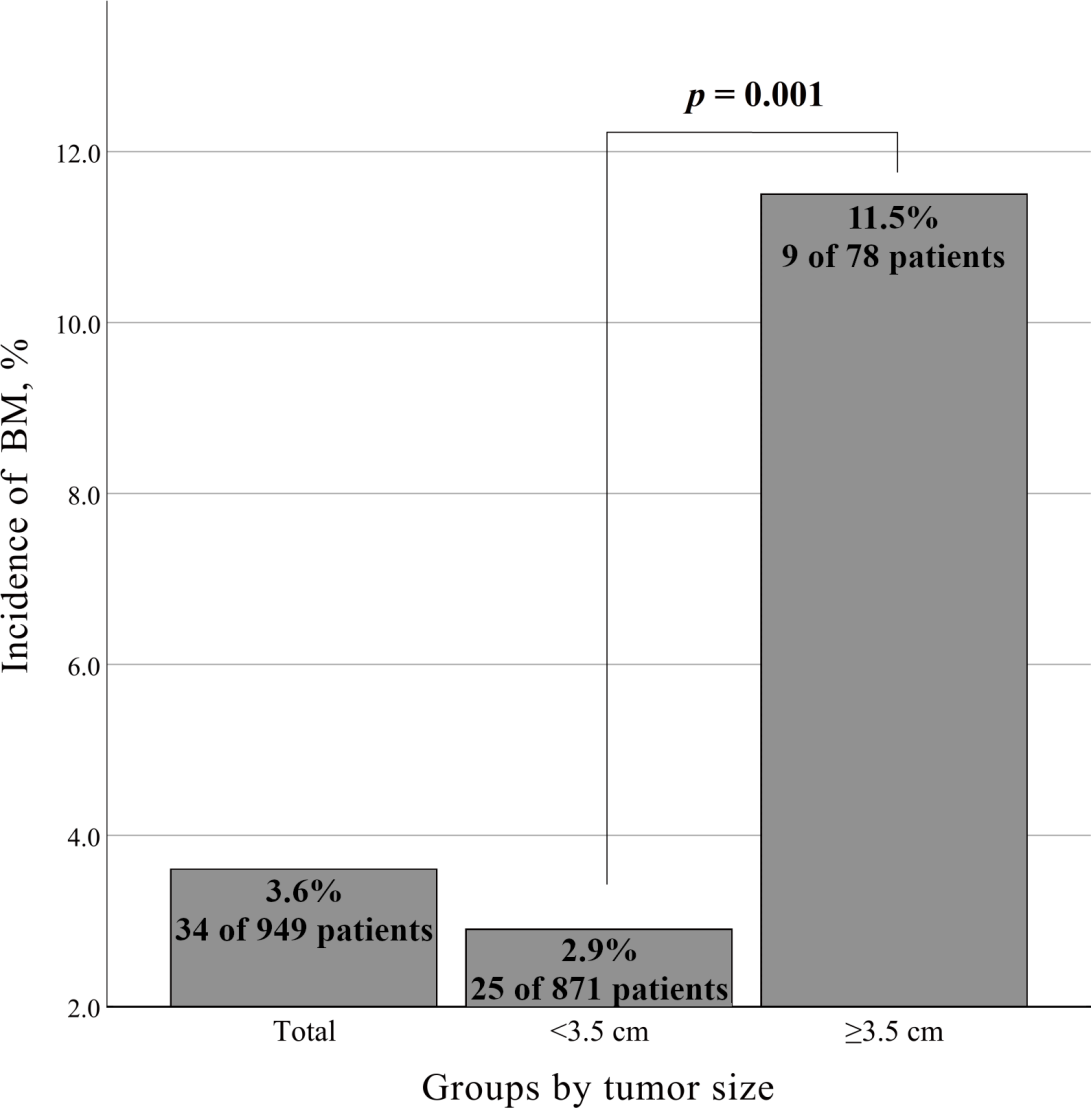
Incidence of brain metastasis according to tumor size.

Firth’s bias-reduced logistic regression model showed that the primary tumor size (OR 1.056; 95% CI 1.009-1.106, p=0.018) was the only predictor of the presence of BM (**Table 3**). Fisher’s exact test with the minimum p value approach was used to determine the optimal cutoff value in tumor size for predicting BM, and a tumor size of 3.5 cm was determined to be the optimal cutoff (**Figure 2**). The incidence of BM in patients with tumor size ≥3.5 cm was 11.5% (9 of 78 participants), as opposed to 2.9% (25 of 871 participants) with tumor size <3.5 cm, and the difference was statistically significant (p=0.001, **Figure 3**).

**Table 3:** Firth’s bias-reduced logistic regression analysis assessing predictors of isolated brain metastasis in participants who underwent brain MRI.

Variables	OR (95% CI)	*p value*
Female	1.113 (0.563-2.199)	0.756
Age, y	1.003 (0.971-1.037)	0.854
Ever-smoker	0.804 (0.402-1.582)	0.527
Adenocarcinoma	1.209 (0.517-3.411)	0.682
Tumor size, mm	1.056 (1.009-1.106)	0.018

OR, odds ratio; CI, confidence interval.

In subgroup analysis (n=818), we also investigated whether epidermal growth factor receptor (EGFR) mutation was associated with increased incidence of brain metastasis in our cohort (**Table S1**). In this analysis, EGFR mutation did not show significant association with incidence of brain metastasis (p >0.05), and the primary tumor size was also the only predictor of the presence of BM (OR 1.100; 95% CI 1.040-1.168, p=0.001).

### Treatment modalities and prognosis of patients with BM

Thirty-four patients with BM were treated with combinations of surgery, stereotactic radiation therapy (SRT), and systemic chemotherapies (**Table S2**). Kaplan–Meier estimation showed that the median overall survival of the patients with BM was 5.5 years, and the 5-year survival rate was 59.8% (**Figure S3**).

### Analysis of differentially expressed genes

**Figure 4:**
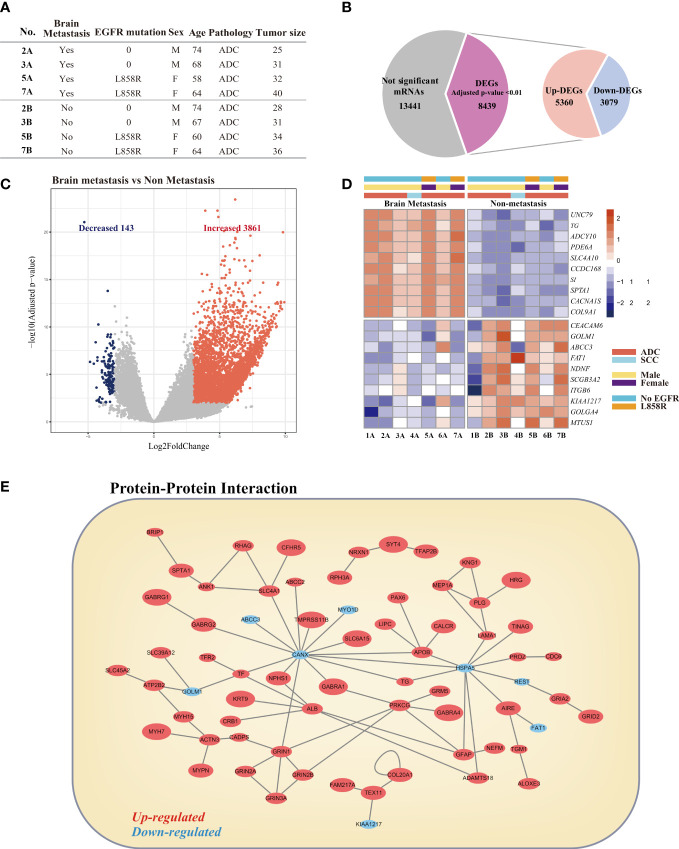
Comparative analysis of differentially expressed genes (DEGs) in lung cancer with brain metastasis and lung cancer without brain metastasis. **(A)** Clinical characteristics of patients selected for analysis. **(B)** Venn diagrams for the DEGs in the two comparison groups. Significance was defined by adjusted p-value <0.01. **(C)** Volcano plot of the DEGs. **(D)** Heatmap visualization of the top 10 most upregulated and top 10 most downregulated DEGs. **(E)** Protein–protein network of DEGs.

Among the 34 patients with BM, 14 patients had paraffin-embedded tissue bank samples of primary lung lesions available, and the tissue samples from 7 patients were confirmed to be suitable for genetic analysis based on an RNA integrity number greater than 7. From among the 14 samples (**Figure S1**, **Table S3**), we selected 4 transcriptomes of lung adenocarcinoma tissues without BM and 4 matched lung adenocarcinoma tissues with BM (**Figure 4A**). Three pairs of transcriptome data (1A/1B, 4A/4B, 6A/6B in **Table S3**) were excluded from the analysis because of mismatches and heterogeneity of baseline characteristics (i.e., ALK mutation in 1A/1B, squamous cell carcinoma in 4A/4B, E746_A750del/E19del mutations in 6A/6B). Then, we analyzed differentially expressed genes (DEGs) in these two groups and identified a total of 8,439 DEGs with adjusted p-value <0.01 (**Figure 4B**). Among these genes, we identified 3,861 upregulated genes and 143 downregulated genes using |log2 fold change| >3 as cutoffs (**Figure 4C**). The top 10 significantly upregulated genes and top 10 significantly downregulated genes are shown in **Figure 4D**. These 20 genes were also significantly differentially expressed between 7 lung adenocarcinoma tissues without BM and 7 lung adenocarcinoma tissues with BM (**Figure 4D**).

To investigate the potential interactions between DEGs and identify proteins that may have critical roles in BM of lung cancer, we constructed a protein–protein interaction (PPI) network using Cytoscape software (16). The PPI network among the DEGs was analyzed based on 84,683 protein–protein interactions for 11,649 proteins obtained from the following five interactome databases-interactome databases: the Biological General Repository for Interaction Datasets (BioGRID) (17), the IntAct molecular interaction database (IntAct) (18), the Molecular INTeraction database (MINT) (19), the Database of Interacting Proteins (DIP) (20), and the Interologous Interaction Database (I2D) (21). The visualized network is shown in **Figure 4E**. The top 5 hub nodes were calnexin (CANX), heat shock protein family A (Hsp70) member 5 (HSPA5), protein kinase C gamma (PRKCG), albumin (ALB), and glutamate ionotropic receptor NMDA type subunit 1 (GRIN1).

### Gene ontology and pathway enrichment analysis of differentially expressed genes

GO analysis showed that DEGs were significantly enriched in three categories of function: biological processes (BP), cellular components (CPs), and molecular function (MF) (**Figure S2A**). The most enriched functions in the BP were related to ‘sensory perception’ and ‘cotranslational protein targeting to membrane and endoplasmic reticulum (ER)’. The most enriched functions in the CPs were related to ‘presynapse’, ‘cell-substrate junction’, and ‘focal adhesion’. The most enriched functions in MF were related to ‘olfactory receptor activity’, ‘passive transmembrane transporter activity’ and ‘channel activity’. KEGG pathway analysis showed that the enriched pathways associated with BM were ‘neuroactive ligand–receptor interaction’, ‘nicotine addiction’, ‘COVID-19’, ‘taste transduction’, and ‘estrogen signaling pathway’ (**Figure S2B**).

### Differentially expressed genes related to brain metastasis, tumor microenvironment and epithelial-mesenchymal transition

**Figure 5:**
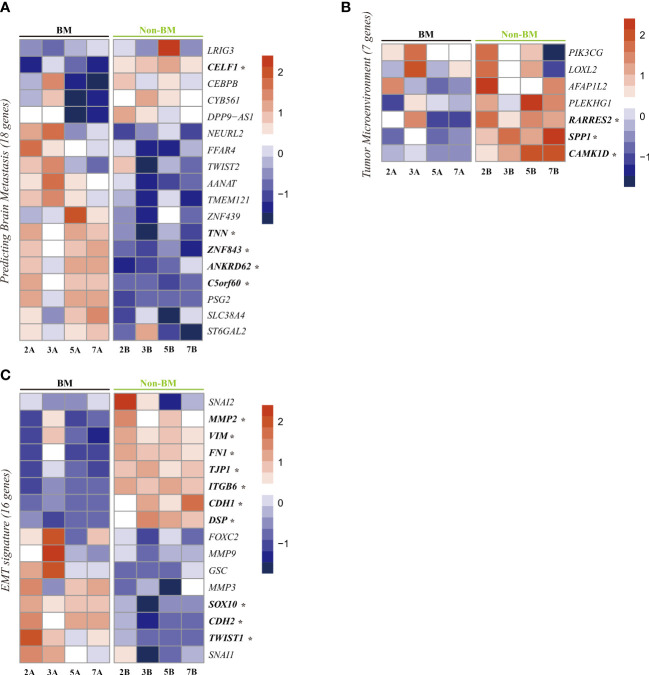
Heatmap visualization of gene expression related to brain metastasis. **(A)**, tumor microenvironment **(B)**, and epithelial-mesenchymal transition **(C)** in lung cancer with brain metastasis and lung cancer without brain metastasis. *Significant DEGs (p-value <0.01).

We next focused on the expression of known genes related to BM. Iris Kamer et al. suggested a set of 22 genes from primary NSCLC tumors as risk genes for BM development after surgical resection of NSCLC (22). We detected the expression of 18 genes in our dataset (**Figure 5A**). Among these 18 genes, five showed significant differences in our DEGs. The significantly upregulated genes were tenascin N (*TNN*), zinc finger protein 843 (*ZNF843*), ankyrin repeat domain 62 (*ANKRD62*), and chromosome 5 open reading frame 60 (*C5orf60*), and the downregulated gene was CUGBP elav-like family member 1 (CELF1).

Regarding the tumor microenvironment (TME), we checked seven known genes related to the TME of lung cancer (23). Among these seven genes, three genes showed significant differences between patients with BM and patients without BM (**Figure 5B**). These three downregulated genes are retinoic acid receptor responder 2 (*RARRES2*), secreted phosphoprotein 1 (SPP1) and calcium/calmodulin-dependent protein kinase 1D (CAMK1D).

Epithelial-mesenchymal transition (EMT) also plays an important role in tumor metastasis (24). To assess the changes in EMT-related genes, we checked 16 genes known to be related to EMT (25) in our dataset. Among these genes, three epithelial marker genes (TJP1, CDH1, DSP) were upregulated in patients without BM, and three EMT driver genes (SOX10 (26), TWIST1 (14), CDH2 (27)) were upregulated in patients with BM (**Figure 5C**).

### NALCN inhibition suppresses lung cancer cell proliferation and migration

The UNC79 (unc-79 homolog) gene encodes the UNC79 protein, which functions as an accessory subunit of the NALCN complex subunit (28). Among the DEGs involved in BM, *UNC79* was the most highly expressed gene in the lung adenocarcinoma tissues from the BM group (**Figure 4D**). To investigate the role of NALCN in lung cancer proliferation and metastasis, we performed an *in vitro* assay using A549 cells with the NALCN inhibitor L-703,606 (29).

**Figure 6:**
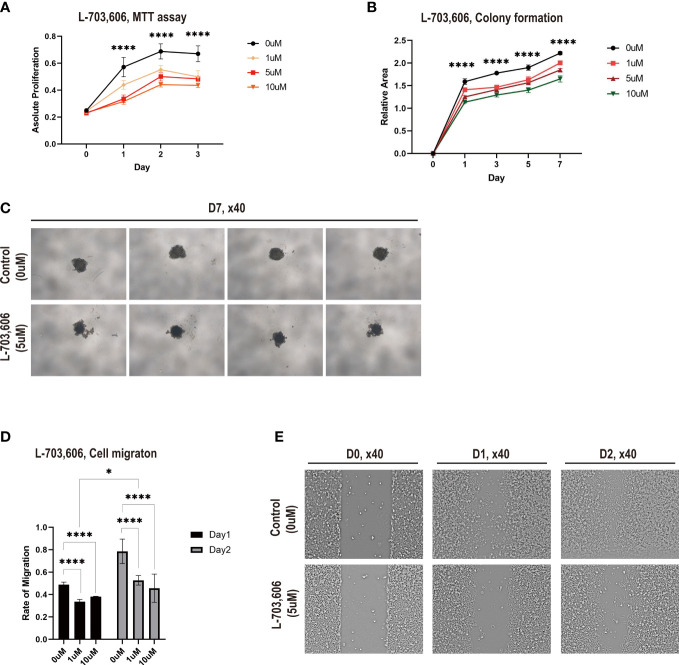
Na+ leak channel, non-selective (NALCN) inhibition suppresses lung cancer cell proliferation and migration. **(A)** Cell proliferation was detected by MTT assay following L-703,606 treatment of A549 cells. The experiment was repeated 3 times. **(B)** Colony formation of A549 cells treated with L-703,606. The experiment was repeated 2 times. **(C)** Representative images of the colony formation assay. The experiment was repeated 3 times. **(D)** Wound healing assay of A549 cells treated with L-703,606. **(E)** Representative images of the wound healing assay. **** and * represent the difference is significant at a significance level of 0.05.

NALCN inhibitor administration in A549 cell lines inhibited cell proliferation. According to the MTT assay, L-703,606 (1 to 10 µM) in A549 cells significantly inhibited cell growth at day 1, day 2, and day 3 (**Figure 6A**). More specifically, the rate of inhibition by L-703,606 on each day was 20~24% with 1μM, 28~42% with 5μM and 36~45% with 10μM. The experiment was repeated 3 times.

Then, to explore the effects of NALCN inhibition on lung cancer cytotoxicity, we performed a cell survival assay based on colony formation. **Figures 6B, C** demonstrate that L-703,606 significantly reduced colony formation after 7 days (**Table S4**). This experiment was repeated 2 times.

The effects of NALCN inhibition on A549 cell migration capability were assessed by a wound-healing assay. In the wound-healing assay (**Figures 6D, E**), L-703,606 (1 to 10 µM) in A549 cells significantly decreased wound closure rates at day 1 and day 2. The disparity of migration on day 1 was 31% with 1μM and 22% with 10μM, and the disparity of migration on day 2 was 32% with 1μM and about 42%. This experiment is repeated with 3 times. These data suggest that NALCN inhibitors have effective tumor cell toxicity against lung cancer cells and may suppress lung cancer metastasis.

## Discussion

### Incidence of BM in suspected early-stage NSCLC

Patients with suspected early-stage NSCLC based on PET scans may still have BM. If brain MRI evaluation is not considered in these patients, they will be misdiagnosed as stage I and be offered suboptimal treatment, which may be associated with poor prognosis. This study investigated the clinical features and genetics of BM in patients with suspected early-stage NSCLC to establish a better ground for using brain MRI to screen these patients.

In our study, the incidence of BM in patients with suspected early-stage NSCLC was 3.6% overall, and the incidence increased to 11.5% in patients with a primary tumor size ≥3.5 cm. To our knowledge, the specific incidence of BM according to primary tumor size has not been well documented in previous studies, especially in patients who were thoroughly prescreened with PET scans to exclude distant metastases other than BM.

Milano et al. investigated the incidence of BM in patients with node-negative NSCLC with the Surveillance, Epidemiology, and End Results (SEER) registry data of 49,495 participants with stage T1-3N0 disease (11). In that study, the incidence of BM in patients with T1N0 clinical staging was revealed to be approximately 3%, which was similar to our results. However, the previous study did not exclude patients with known distant metastasis other than BM, and data regarding the diagnostic method used for staging were not available. In our study, all the participants underwent PET scans to exclude distant metastases other than BM, and the presence of BMs was confirmed by contrast-enhanced brain MRI.

### Clinical risk factors for BM in suspected early-stage NSCLC

A limited number of previous studies have suggested a few clinical risk factors for BM at initial presentation in patients with NSCLC (11, 30–32). Younger age (11, 31, 32), nonsquamous histology (11, 31, 32), tumor size (11, 30–32), tumor grade (11, 31, 32), and advanced node stage (11, 30–32) were clinical factors associated with BM. However, those previous studies did not exclude patients with distant metastasis other than BM. Therefore, the effects of previously suggested risk factors may not be specific for metastasis to the brain, and the confounding effect of distant metastasis in general and the associated increased tumor burden could not be excluded. Therefore, the clinical implications of the results of previous studies might be limited in terms of evaluating risk factors for BM in suspected early-stage NSCLC.

In our study, the only relevant clinical risk factor for BM was tumor size, and other clinical factors (i.e., sex, age, smoking history, tumor pathology, EGFR mutations) did not show significance as risk factors regarding BM in the participants. These results are similar to those of a previous study (33) conducted by Zhuge et al., which excluded patients with distant metastasis other than BM. The study showed a correlation between tumor size and the incidence of brain metastasis, but other clinical factors were not associated with an increased risk of BM. However, the study did not adopt PET as a routine staging procedure, which might limit the general applicability of its conclusions.

In particular, an analysis of our cohort with the minimum p value approach determined the optimal cutoff value in tumor size for predicting BM: tumor size ≥3.5 cm. If routine brain MRI screening is implicated in a subpopulation with suspected early-stage NSCLC, the cutoff value of 3.5 cm in tumor size may be considered the threshold.

### Favorable prognosis of patients with BM in suspected early-stage NSCLC

NSCLC with BM in general often results in a poor prognosis, with a median survival of 4 to 12 months (34, 35). However, the median survival of the patients with BM in our study was 5.5 years, and the 5-year survival rate was 59.8%, which is better than previously reported in the literature. The better prognosis of our study participants may be due to the lower tumor burden without lymph node involvement or other distant metastases and the application of an active treatment approach for each lesion with/without adjuvant chemotherapy. These results suggest a need for a more aggressive screening plan for specific populations because proper diagnosis of BM at initial presentation can assure initiation of treatment at the proper time, which can result in a favorable prognosis.

### Analysis of DEGs related to BM in suspected early-stage NSCLC

It has been shown that, other than TNM staging, the progression and prognosis of tumors are related to high expression of some genes in NSCLC (22, 36). Although a scarcity of clinical predictors for BM was observed in our cohort, we also identified a list of genes that may be biomarkers of BM in those patients.

We selected the top 10 significantly upregulated genes and top 10 significantly downregulated genes, and these 20 genes were significantly differentially expressed between 7 lung adenocarcinoma tissue samples without BM and 7 lung adenocarcinoma tissue samples with BM. To determine the biological relationship and signaling pathways among the 20 mRNAs in the signature, we performed GO and KEGG analyses.

The GO terms were enriched in several important molecular mechanisms, such as cotranslational protein targeting to membrane and ER, focal adhesion, and olfactory receptor activity. These pathways are considered to be closely related to tumors and metastasis (37–39). The main KEGG pathways involved included neuroactive ligand–receptor interactions, which were found to have prominent roles in adapting to the target organ environment in BM (40).

We also identified 3 genes related to TME as significant DEGs (i.e., RARRES2, SPP1, CAMK1D). RARRES2 is a multifunctional, chemoattractant protein known for its roles in angiogenesis and tumorigenesis (41), and previous studies reported that lower levels of RARRES2 expression in tissue were significantly associated with tumor-node metastasis stage, degree of differentiation, and poor survival rates (42, 43). Furthermore, CAMK1D is known as a negative regulator of angiogenesis, and overexpression of CAMK1D in mouse model suppressed neoangiogenesis and expansion of lung tumor by limiting the tumor’s ability to co-opt the alveolar vasculature (44). These findings correlate our current study, which showed significant down-regulation of RARRES2 and CAMK1D in patients with BM.

On the other hand, previous study regarding SPP1 expression in lung cancer generally provides positive association with poor outcomes, which is not concordant with our current study (45, 46). Furthermore, contrary to previous studies, some of genes we investigated in relation to TME (as well as EMT signature) did not show significant difference in expression. However, the previous studies investigated patients with lung cancer in general, which was different population from our study. In our study, all the patients had no lymph node metastasis or distant metastasis other than brain, and they were suspected to have early-stage lung cancer before performing brain MRI. Therefore, our findings may suggest a clue for more specific biomarkers for metastasis to brain in lung cancer, rather than predictors for general prognosis or TNM staging in lung cancer. Further research to investigate this hypothesis is warranted.

EMT is known to play an important role in tumor metastasis (23), and our results showed that three EMT driver genes were upregulated in patients with BM. A previous study (47) demonstrated that EMT is involved in the process of metastatic dissemination to the brain, and the EMT driver genes that were upregulated in our study (SOX10, TWIST1, CDH2) may have potential as biomarkers in risk stratification for BM in suspected early-stage lung cancer.

Previous studies showed that high expression of UNC79 (48), CACNA1S (49), and COL9A1 (50) was correlated with metastasis-associated tumor behaviors, and patients with BM in our study also showed high expression of those genes. On the other hand, the FAT1 (51), NDNF (52), and MTUS1 (53) genes, which were shown to be related to tumor suppressive properties in previous studies, showed significantly lower expression levels in patients with BM in our study.

Among those candidate genes, UNC79, which is the subunit of the NALCN channel complex and the top upregulated gene in patients with BM, was evaluated in the present study. An *in vitro* assay using A549 cells revealed that the NALCN inhibitor suppressed lung cancer cell proliferation and migration. This finding suggests that UNC79 have a potential to be a biomarker for advanced lung cancer, although we cannot confirm that UNC79 is specifically associated with brain metastasis. However, if those candidate genes can be applied as biomarkers in specific population as in our study (i.e., suspected early-stage NSCLC based on CT and PET scans), targeted strategy for screening brain metastasis may still be feasible in the future. Further research with larger cohort to explore this hypothesis is warranted.

Taken together, our findings demonstrate a multiple-mRNA signature that might be closely related to BM in suspected early-stage lung cancer and may aid in the identification of novel biomarkers for predicting BM in those populations.

## Limitations

Our study has some limitations. First, it was a single-center study with a retrospective design. However, our study was carefully designed to evaluate the clinical efficacy of brain MRI screening for specific populations, and all the participants were screened with PET and contrast-enhanced brain MRI, which was a rarely adopted methodology in previous studies. Second, no validation cohort was available to support our observations on correlations between tumor sizes and presence of brain metastasis. Third, we performed transcriptome analysis on RNA extracted from formalin-fixed paraffin-embedded (FFPE) tissues from patients. Although we used an optimized method for RNA sequencing from FFPE tissues, our results may differ from the transcriptome of fresh lung cancer tissues. Besides, a small number of available tissue samples is a limitation that should be clearly acknowledged. However, recent study suggested that RNA-sequencing analysis using DESeq2 method performs well when the sample size equals to 3 in each group, with relatively better FDR control and higher power than most of other methods (54). In that sense, we adopted DESeq2 method, and we also set adjusted p-value <0.01 to compensate the limitation of small samples. Clearly, more research in larger cohorts with fresh lung cancer tissues is needed to validate our findings.

## Conclusions

Given the incidence and favorable outcome of BM in patients with suspected early-stage NSCLC, selective screening with brain MRI should be considered, especially in patients with high-risk features.

## Data availability statement

The datasets presented in this study can be found in online repositories. The names of the repository/repositories and accession number(s) can be found here: (https://www.ncbi.nlm.nih.gov/bioproject/?term=) and can be accessed using the accession number PRJNA932864.

## Ethics statement

The studies involving human participants were reviewed and approved by Ethics Review Committee of Severance Hospital (IRB No. 4-2020-1306). Written informed consent for participation was not required for this study in accordance with the national legislation and the institutional requirements.

## Author contributions

KK contributed to conception, data acquisition, design, analysis, and writing. JL, J-YL contributed data acquisition and analysis. SY, EK, JJ, YAK, MP, YSK contributed to data acquisition and writing. C-MO and SL contributed to funding, conception, data acquisition, design, and writing. All authors contributed to the article and approved the submitted version.
