# Supplementary material for: Clinical features and molecular genetics associated with brain metastasis in suspected early-stage non-small cell lung cancer
Source: Front Oncol. 2023 Apr 17;13:1148475. doi: 10.3389/fonc.2023.1148475 (PMC10150586; doi:10.3389/fonc.2023.1148475)
Supplement: Supplementary file 1 [file DataSheet_1.docx]

**Clinical features and molecular genetics associated with brain metastasis in suspected early-stage non-small cell lung cancer**

Kangjoon Kim^1^, Jibeom Lee^2^, Jeong-Yun Lee^2^, Seung Hyun Yong^1^, Eun Young Kim^1^, Ji Ye Jung^1^, Young Ae Kang^1^, Moo Suk Park^1^, Young Sam Kim^1^, Chang-Myung Oh^2,*^, Sang Hoon Lee^1,*^

^1^ Division of Pulmonary and Critical Care Medicine, Department of Internal Medicine, Severance Hospital, Yonsei University College of Medicine, Seoul, Republic of Korea

^2^ Department of Biomedical Science and Engineering, Gwangju Institute of Science and Technology, Gwangju, Republic of Korea

**^*^Correspondence should be addressed to:**

Chang-Myung Oh, MD, PhD

Tel: +82-10-7304-1213

E-mail: cmoh@gist.co.kr

ORCID ID: **0000-0001-6681-4478**

Sang Hoon Lee, MD, PhD

Tel: +82-2228-1930

E-mail: cloud9@yuhs.ac

ORCID ID: 0000-0002-7706-5318

**Supplementary Materials**

**Supplementary Methods**

RNA-sequencing Differential Expression Analysis

The quality of sequence reads was verified using FASTQC (version 0.11.5), and the adaptor sequence and low-quality bases were eliminated using Cutadapt (version 2.10), and trimmed reads were mapped using STAR (version 2.7.3a) to human reference genome. Aligned sequences were quantified using HTSeq-count (version 0.6.1p1) by Ensembl ID. Raw count data were normalized by a median of ratio method in size of patient samples and transformed using regularized log transformation function in DESeq2 packages. Then, gene expression of specific groups was calculated as z-score, and the visualization was performed using pheatmap packages (version 1.0.12) in R (version 4.1.0).

For differential expression analysis, we included the transcriptome of 7 patients as the analysis groups according to two features, single brain metastasis or non-metastasis and having EGFR L858R mutation or not, except other types of mutation. Raw counts matrix of selected 7 patients was filtered by which the gene expression of all analysis sample needs over 70% of total sum.

Pre-filtered counts were provided as input data for DESeqDataSetFromMatrix function of DESeq2 packages (version 1.32.0) into R (version 4.1.0) with two models, brain metastasis and EGFR mutation. The results of Differential expression analysis were extracted by DESeq and results function. After DEGs, gene names by Ensembl ID were altered by NCBI Entrez ID using biomart packages in R, no-matched genes were removed in proceeding analysis, as results 21880 genes.

With false discovery rate (FDR) adjusted p-value cutoff as 0.01, the number of genes was separated by the positive and negative value of log2 fold change, and the detailed cutoff value for log2 fold change was described in volcanoplot.

Pathway enrichment analysis

For pathway analysis, ClusterProfiler packages (version 4.0.5) indicated the pathway enrichment in terms of gene ontology (GO) and Kyoto Encyclopedia of Genes and Genomes (KEGG). GO and KEGG datasets were accessed by DOSE. The input data for the enrichment pathway was the value of log2 fold change with FDR adjusted p-value < 0.01, labeled as Entrez ID. The statistical method for p-value adjustment was Benjamini-Hochberg, and the p-value cutoff is 0.05.

In a case which various pathways are estimated in results, we described the maximum 20 and 40 pathways, arranged by the lowest adjusted p-value in each GO pathway analysis and KEGG pathway. Protein-protein interaction was analyzed by Cytoscape. (version 3.8.2).

Cell culture and proliferation

The adenocarcinomic human alveolar basal epithelial A549 cells were cultured in Roswell Park Memorial Institute (RPMI)-1640 medium supplemented with 10% fetal bovine serum (FBS) and 1% penicillin/streptomycin (P/S) in a humidified atmosphere of 5% CO_2_ at 37℃.

Cell proliferation assay

Cell proliferation was assessed by through the method of staining of living cells with MTT (3- [4,5-dimethylthiazol-2-yl] −2,5-diphenyltetrazolium bromide). The A549 cells were seeded in 96-well plate about 12,000 cells in each well. The cells were incubated for 1 day. Using the dimethyl sulfoxide (DMSO, Sigma Aldrich) and RPMI-1640 medium, L-703,606 oxalate salt (Sigma Aldrich) were solved in concentration of 1μM, 5μM and 10μM. The solution is composed with 1% DMSO and 99% RPMI-1640 medium (contained with 10% FBS and 1% P/S). The medium of 96-well plate was replaced every day using the solution. After 24 h, 48 h, and 72 h of L-703,606 incubation, MTT assay was performed using Cell Proliferation Kit Ⅰ (MTT) (purchased from Roche) according to the manufacturer’s protocol.

*In vitro* wound healing assay

A549 cells were seeded in 12-well plate about 1ⅹ10^6^ cell/mL in each well with 1mL RPMI medium with 10% FBS and 1% P/S. The cells were incubated for 1 day at least. A straight line was scratched the center of well using a 200uL sterile plastic pipette tip. After, we washed the well using the PBS gently. Then, we put 1mL RPMI-1640 medium with the chemicals (L-703,606 oxalate salt hydrate) which were solved in DMSO. Concentration of chemicals was 1μM, 5μM and 10μM and maximum concentration of DMSO was 1% (volume rate). The images of migration were taken after 0h, 24h and 48h.

Colony formation assay

A549 cells were seeded in 96-well spheroid plate about 1000 cells in each well. The concentration of chemical (L-703,606 oxalate salt hydrate) was 1μM, 5μM and 10μM with RPMI-1640 medium and DMSO. Then, we added the same condition of medium with 100μL every 2 days without sucking medium. The images of colony formation were taken after 0h, 24h, 72h, 120h and 168h.

**Supplementary Figures**

Figure S1. Clustering visualization of RNA-sequencing data. (A) Heatmap of distance matrix. Color intensity represents sample distance. (B) Principal component analysis plot. Orange cluster represents the sample with brain metastasis. Blue cluster represents the sample without brain metastasis.


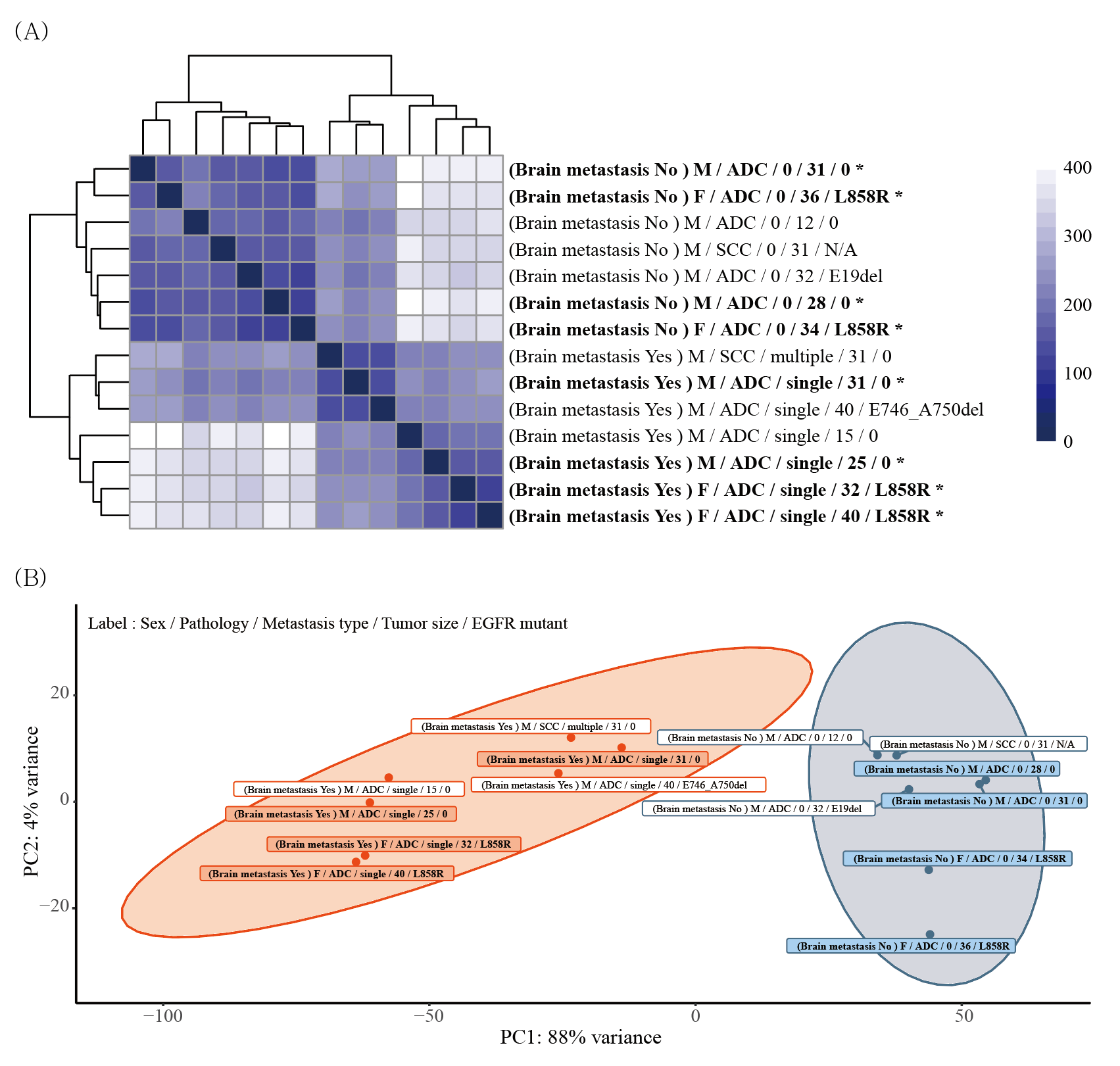


Figure S2. Significantly enriched pathways of DEGs. (A) Significantly different gene ontology biological processes of DEGs. (B) Enrichment analysis of the Kyoto Encyclopedia of Genes and Genomes pathway.


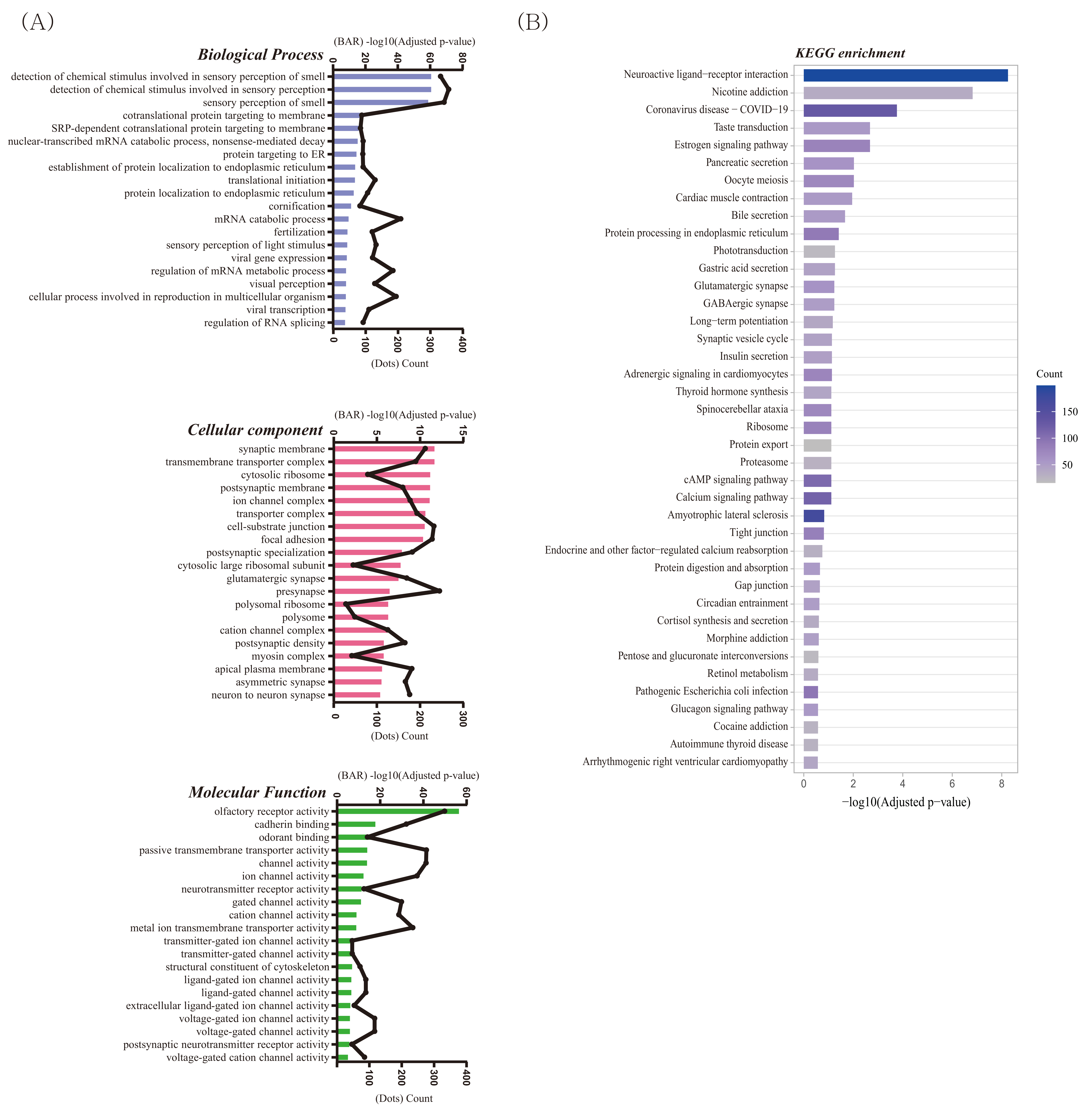


Figure S3. Kaplan-Meier curve displaying the estimated survival probability of patients with isolated brain metastasis (n=34)


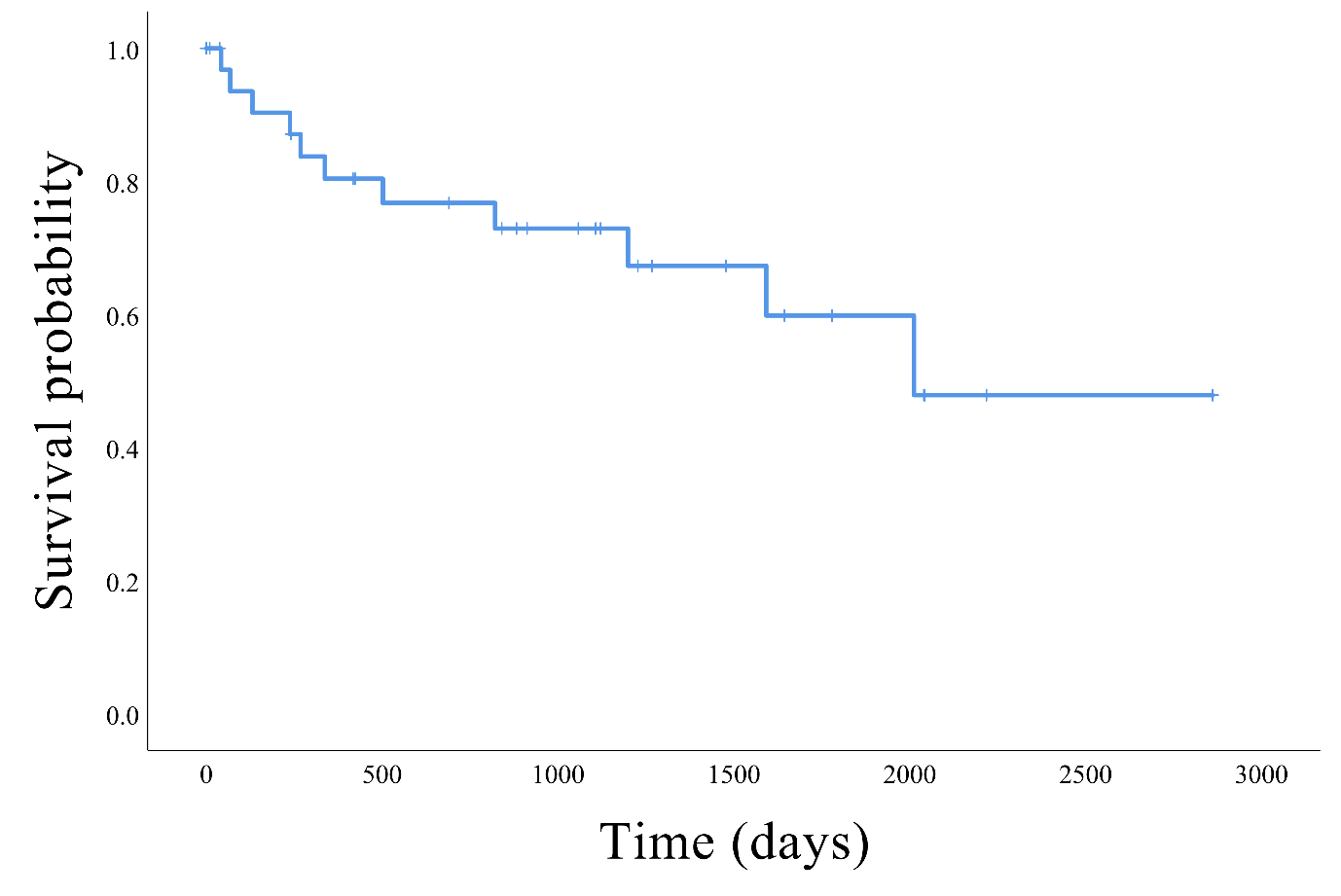


**Supplementary Tables**

Table S1. Subgroup analysis of patients who tested for EGFR mutations (n=818) assessing predictors of isolated brain metastasis. Firth’s bias-reduced logistic regression analysis was adopted for this analysis.

| Variables | OR (95% CI) | *p value* |
| --- | --- | --- |
| Female | 0.458 (0.186-1.060) | 0.068 |
| Age, y | 0.978 (0.939-1.019) | 0.290 |
| Ever-smoker | 2.253 (0.978-5.294) | 0.056 |
| Adenocarcinoma | 0.315 (0.095-1.621) | 0.145 |
| Tumor size, mm | 1.100 (1.040-1.168) | 0.001 |
| EGFR-mutant | 0.572 (0.239-1.317) | 0.190 |

EGFR, epidermal growth factor receptor; OR, odds ratio; CI, confidence interval

Table S2. Case series characteristics of 34 patients with exclusive brain metastasis

| **Patient No.^*^** | **Sex** | **Age, y** | **Ever-smoker** | **Pathology** | **Size, mm** | **First-line treatment** | | |
| --- | --- | --- | --- | --- | --- | --- | --- | --- |
|  |  |  |  |  |  | **Lung** | **Brain** | **Systemic** |
| 1 | F | 56 | - | ADC | 19.5 | Lobectomy with MLND | WBRT | - |
| 2 | F | 60 | - | ADC | 31.1 | - | WBRT | TKI |
| 3 | F | 81 | + | ADC | 34.0 | - | SRS | - |
| 4 | M | 72 | - | ADC | 23.0 | SRT | WBRT | Cytotoxic |
| 5 | F | 55 | - | ADC | 38.0 | - | SRT | TKI |
| 6 | F | 62 | - | ADC | 35.0 | - | SRT | Cytotoxic |
| 7 | F | 46 | - | ADC | 36.0 | - | WBRT | Cytotoxic |
| 8 | F | 58 | - | ADC | 26.0 | - | SRT | TKI |
| 9 | F | 54 | - | ADC | 38.3 | Lobectomy with MLND | - | Cytotoxic |
| 10 | M | 44 | - | ADC | 24.0 | - | SRS | Cytotoxic |
| 11 | M | 52 | + | SqCC | 26.0 | - | SRS | Cytotoxic |
| 12 | F | 80 | - | ADC | 27.0 | - | SRT | TKI |
| 13 | F | 79 | - | ADC | 37.0 | N/A | N/A | N/A |
| 14 | F | 72 | - | ADC | 20.0 | Lobectomy with MLND | - | - |
| 15 | M | 71 | + | ADC | 38.0 | Lobectomy with MLND | SRS and craniotomy | - |
| 16 | M | 68 | + | SqCC | 20.0 | Lobectomy with MLND | - | Cytotoxic |
| 17 | M | 62 | + | ADC | 18.0 | Lobectomy with MLND | - | Cytotoxic |
| 18 | F | 58 | - | ADC | 18.4 | Lobectomy with MLND | - | - |
| 19 | F | 56 | - | ADC | 13.0 | Lobectomy with MLND | - | TKI |
| 20 | M | 56 | + | ADC | 17.7 | SRT | SRS | Cytotoxic |
| 21 | F | 77 | - | ADC | 21.0 | - | SRS | Cytotoxic |
| 22 | M | 62 | + | ADC | 35.0 | - | SRS | Cytotoxic |
| 23 | M | 76 | + | ADC | 16.0 | Lobectomy with MLND | SRS | TKI |
| 24 | M | 88 | - | ADC | 23.5 | SRT | - | - |
| 25 | M | 77 | + | ADC | 17.0 | - | - | TKI |
| 26 | M | 79 | + | SqCC | 37.0 | SRT | WBRT | - |
| 27 | F | 75 | - | ADC | 37.4 | - | - | TKI |
| 28 | M | 56 | + | SqCC | 21.2 | Lobectomy with MLND | SRS | Cytotoxic |
| 29 | F | 63 | - | ADC | 6.3 | Segmentectomy | - | - |
| 30 | F | 44 | - | ADC | 28.0 | Lobectomy with MLND | SRS | TKI |
| 31 | M | 72 | + | ADC | 26.5 | - | SRS | TKI |
| 32 | M | 57 | + | SqCC | 22.0 | Wedge resection | SRS | Cytotoxic |
| 33 | M | 74 | + | ADC | 25.0 | Lobectomy with MLND | - | Cytotoxic |
| 34 | M | 75 | + | ADC | 33.0 | N/A | N/A | N/A |

ADC, adenocarcinoma; MLND, mediastinal lymph node dissection; WBRT, whole brain radiotherapy; TKI, tyrosine kinase inhibitor; SRS, stereotactic radiosurgery; SRT, stereotactic radiotherapy; SqCC, squamous cell lung cancer

**^*^** Listed by chronological order based on date of diagnosis

| No. | Case/Control | Sex | Age | Pathology | Tumor size (mm) | Pathologic N stage | EGFR mutation | ALK mutation | ROS1 mutation |
| --- | --- | --- | --- | --- | --- | --- | --- | --- | --- |
| 1A | Case | M | 76 | ADC | 15 | N0 | - | + | - |
| 1B | Control | M | 73 | ADC | 12 | N0 | - | + | **-** |
| 2A | Case | M | 74 | ADC | 25 | N0 | - | - | - |
| 2B | Control | M | 74 | ADC | 28 | N0 | - | - | - |
| 3A | Case | M | 68 | ADC | 31 | N0 | - | - | - |
| 3B | Control | M | 67 | ADC | 31 | N0 | - | - | - |
| 4A | Case | M | 91 | SqCC | 31 | N/A | - | - | - |
| 4B | Control | M | 91 | SqCC | 31 | N0 | N/A | N/A | N/A |
| 5A | Case | F | 58 | ADC | 32 | N0 | L858R | - | - |
| 5B | Control | F | 60 | ADC | 34 | N0 | L858R | - | - |
| 6A | Case | M | 57 | ADC | 40 | N/A | E746_A750del | - | - |
| 6B | Control | M | 68 | ADC | 32 | N0 | E19del | - | - |
| 7A | Case | F | 64 | ADC | 40 | N0 | L858R | - | - |
| 7B | Control | F | 64 | ADC | 36 | N0 | L858R | - | **-** |

Table S3. Clinical and pathological data for the investigated cases.

ADC, adenocarcinoma; SqCC, squamous cell carcinoma

Table S4. Result of cell survival assay based on colony formation. The table shows the rate of decrease in colony number.

| Day  Concentration | Day1 | Day3 | Day5 | Day7 |
| --- | --- | --- | --- | --- |
| 1μM | 11% | 17% | 14% | 10% |
| 5μM | 21% | 20% | 18% | 17% |
| 10μM | 28% | 27% | 26% | 26% |
